# Simultaneous treatment of temporomandibular joint ankylosis with severe mandibular deficiency by standard TMJ prosthesis

**DOI:** 10.1038/srep45271

**Published:** 2017-03-24

**Authors:** YiHui Hu, LuZhu Zhang, DongMei He, Chi Yang, MinJie Chen, ShanYong Zhang, Hui Li, Edward Ellis III

**Affiliations:** 1 Department of Oral Surgery, Shanghai Ninth People’s Hospital, College of Stomatology, Shanghai Jiao Tong University School of Medicine, Shanghai, Key Laboratory of Stomatology, Shanghai, 200011, China; 2 Department of Oral and Maxillofacial Surgery, University of Texas Health Science Center at San Antonio, TX, USA

## Abstract

Temporomandibular joint (TMJ) ankylosis is a refractory disease that is difficult to predictably treat. This study evaluated the prognosis of using standard alloplastic TMJ prostheses for the treatment of TMJ ankylosis in Chinese patients with severe mandibular deficiency. Patients treated from 2013 to 2015 were reviewed. The computer-aided design and manufacture (CAD/CAM) technique was used to guide bony mass removal and locate the TMJ prosthesis (Biomet, USA). Eleven patients were included in this study. All prostheses were successfully installed and stabilized intraoperatively. In 4 patients with severe mandibular deficiency, their mandibular ramus was elongated by the TMJ prosthesis and 2 patients were combined with Le Fort I osteotomy guided by digital templates. Their mean chin advancement was 10.19 mm. Their SNB and ramus heights were also significantly improved after operation (P < 0.05). There was no prosthesis loosening, breakage, or infection leading to removal after a mean follow-up period of 22 months (range, 12-31mos.). Mouth opening was significantly improved from 5.5 mm preoperatively to 31.5 mm postoperatively. TMJ reconstruction with standard alloplastic prosthesis is a reliable treatment for ankylosis, especially in recurrent cases. By CAD/CAM technique, it can correct jaw deformities simultaneously and produce stable results.

Temporomandibular joint (TMJ) ankylosis is characterized by restricted mouth opening. Patients suffering from TMJ ankylosis during their growing period may develop severe jaw deformities with the possibility of obstructive sleep apnea syndrome (OSAS). TMJ ankylosis is very difficult to treat and recurrent ankylosis is common. The classification of TMJ ankylosis is a reasonable guide for choosing treatment methods^1^. According to Yang’s classification, on the basis of coronal computed tomography (CT) reconstruction in 2006^2^, there were two types of ankylosis: those with or without residual condyles. In ankylosis with residual condyles presented on the medial side of the bony fusion, the residual condyle should be kept during the operation and only the lateral bony fusion should be removed (TMJ lateral arthroplasty). This is especially important for growing children because the preserved condyles can develop and remodel later^3^,^4^,^5^. For TMJ ankylosis without residual condyles, joint reconstruction by autogenous bone grafts, alloplastic TMJ prostheses, or distraction osteogenesis should be used after sufficient removal of the bony fusion. In recent years, alloplastic TMJ prostheses have been widely used in Europe and North America. These are stable without resorption compared with autogenous bone grafts. Although there are some reports on the application of ankylosis^6^,^7^,^8^,^9^, most of them are custom ones which are much easier to implant intraoperatively than standard ones. In order to make the bone fit the prostheses accurately, we used computer-assisted technique to guide bone trimming and implantation of the Biomet standard prostheses^10^,^11^,^12^,^13^.

The aim of this study was to evaluate the use of standard alloplastic TMJ prostheses for the treatment of TMJ ankyloses with severe jaw deformity in Chinese patients.

## Results

**Figure 1:**
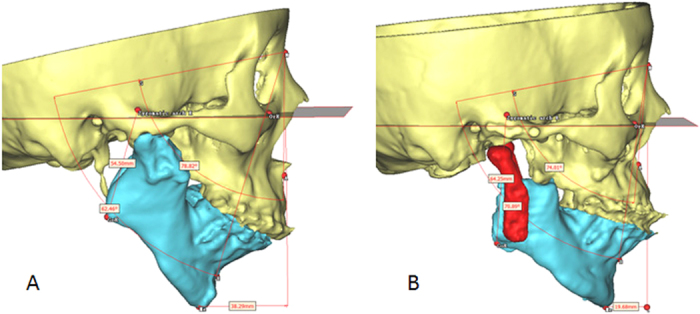
3D measurement of jaw bone deformities (Patient 4). (**A**) Before operation. (**B**) After operation.

**Figure 2:**
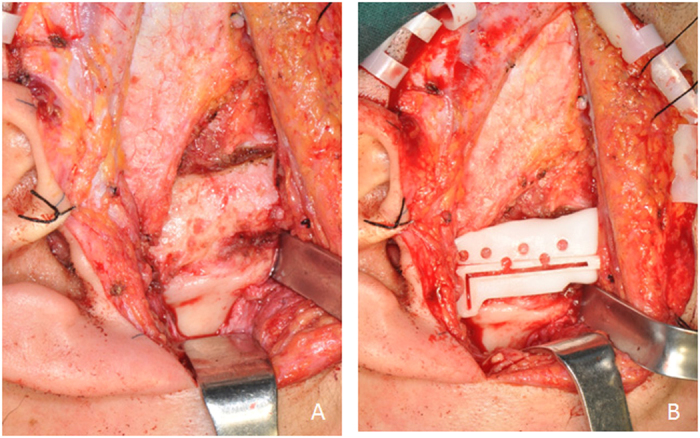
Digital guide to perform osteotomy and glenoid fossa prosthesis placement (Patient 7). (**A**) Exposure of bony fusion by preauricular incision. (**B**) Osteotomy using digital guide and placement of glenoid fossa prosthesis.

**Figure 3:**
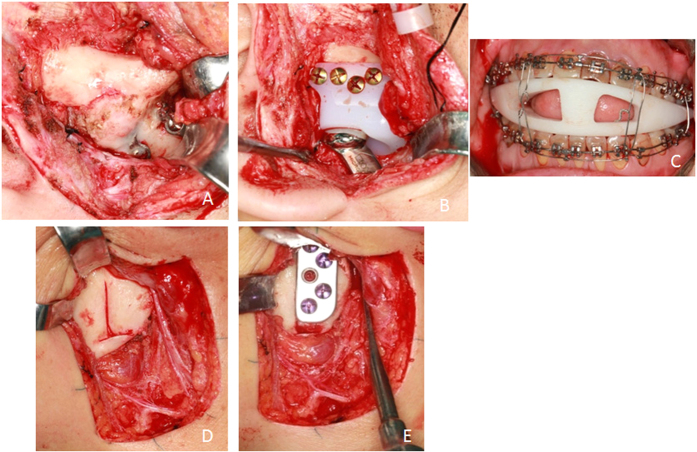
Surgery of recurrent joint ankylosis combined with jaw deformity (Patient 4). (**A**) Recurrent bony fusion revealed by preauricular incision; (**B**) Implantation of glenoid fossa prosthesis; (**C**) Intermaxillary fixation using digital occlusal splint; (**D**) Exposure of ramus by submandibular incision; (**E**) Implantation of mandibular prosthesis.

**Figure 4:**
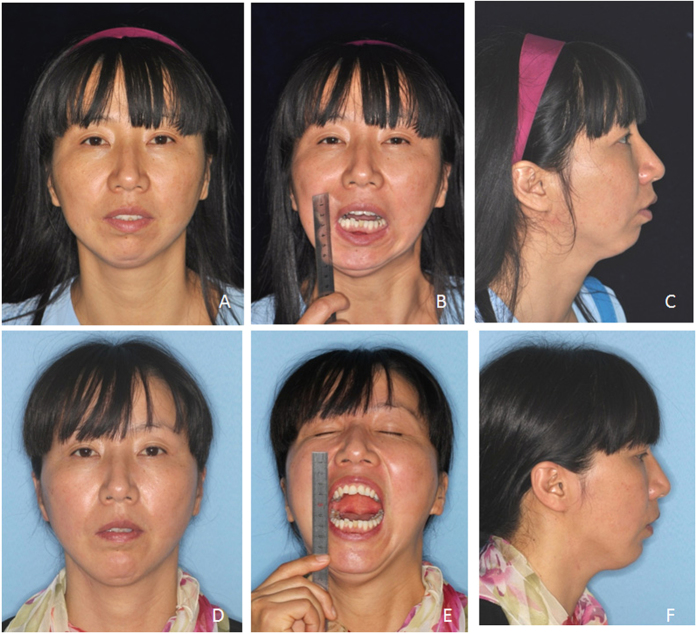
Patient 7, female, 39 yrs, conditions of mandibular retrusion and right TMJ ankylosis before and after surgery. (**A**) Pre-operative frontal view; (**B**) Pre-operative mouth opening view; (**C**) Pre-operative profile view; (**D**) Post-operative frontal view after 23 months; (E) Post-operative mouth opening view; (**F**) Post-operative profile view.

**Figure 5:**
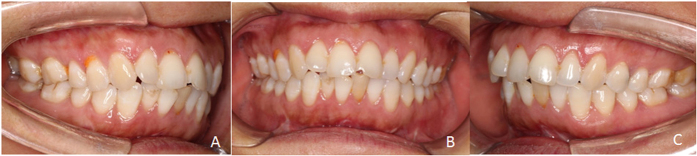
Stable occlusion after operation (Patient 7). (**A**) Right lateral view at maximal intercuspid position; (**B**) Anterior view at maximal intercuspid position; (**C**) Left lateral view at maximal intercuspid position.

**Figure 6:**
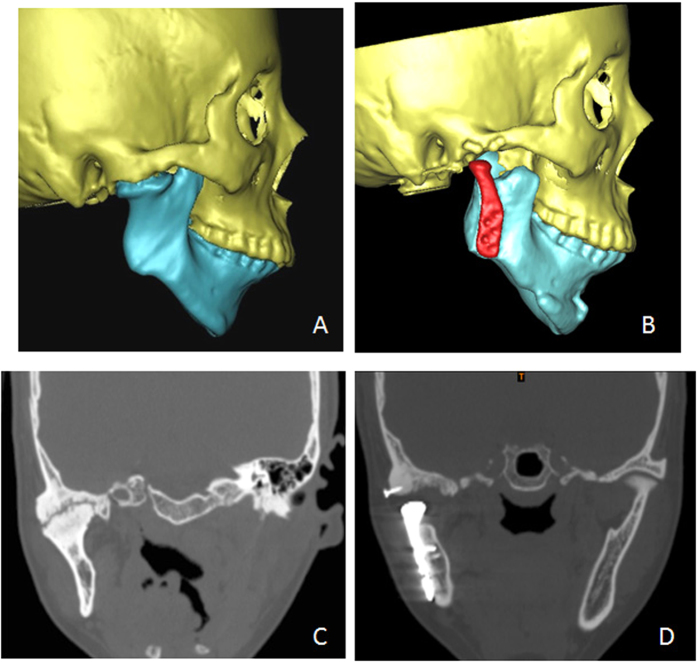
CT images of Patient 7. (**A**) Pre-operative 3D CT; (**B**) Post-operative 3D CT; (**C**) Pre-operative CT coronal reconstruction; (**D**) After 23 months, no relapse of ankylosis can be seen in CT coronal reconstruction.

**Figure 7:**
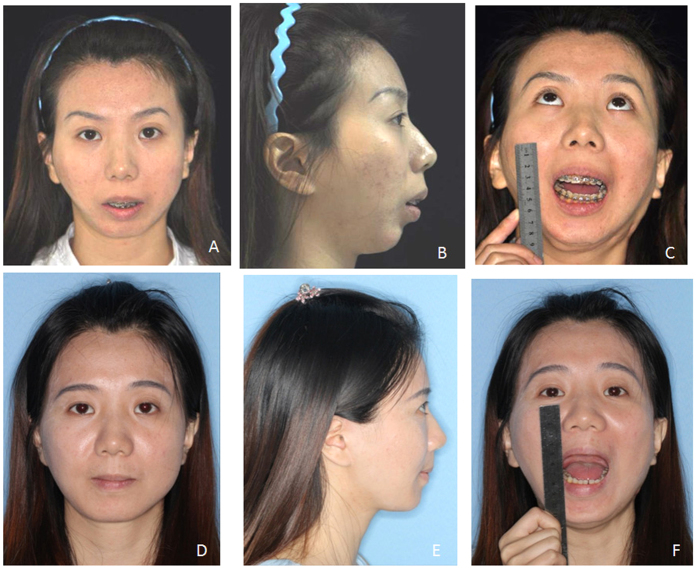
Patient 4, female, 32 yrs, relapse of right TMJ ankylosis after coronoid process implantation with chin retrusion. (**A**) Pre-operative frontal view; (**B**) Pre-operative profile view; (**C**) Pre-operative mouth opening view; (**D**) Post-operative frontal view after 28 months; (**E**) Post-operative profile view; (**F**) Post-operative mouth opening view.

**Figure 8:**
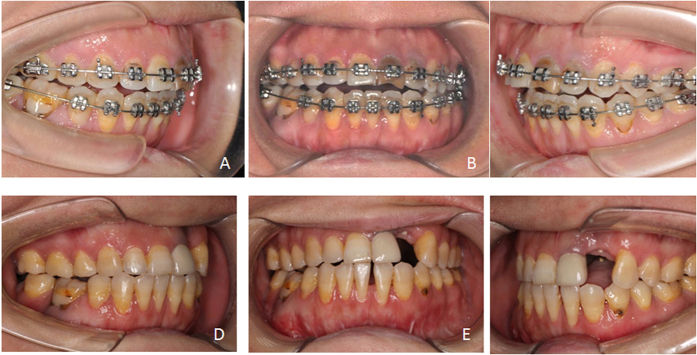
Occlusion before and after operation (patient 4). (**A**) Pre-operative right lateral view at maximal intercuspid position; (**B**) Pre-operative frontal view; (**C**) Pre-operative left lateral view at maximal intercuspid position; (**D**) Post-operative right lateral view at maximal intercuspid position; (**E**) Post-operative frontal view; (**F**) Post-operative left lateral view at maximal intercuspid position.

**Figure 9:**
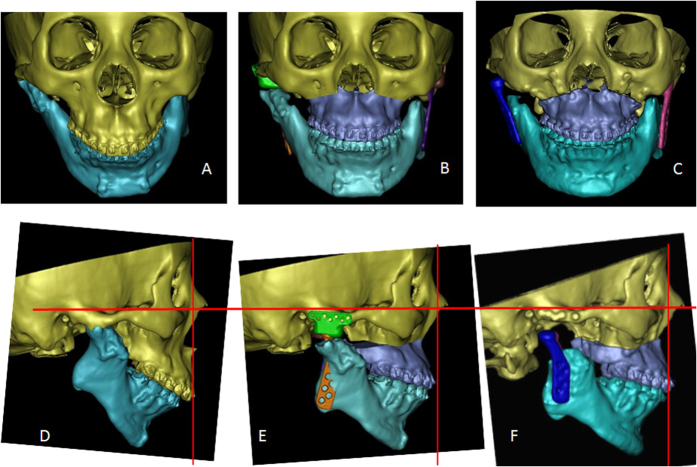
Pre-operative and post-operative CT images of Patient 4. (**A**) Pre-operative frontal view of 3D CT; (**B**) Pre-operative design of frontal view of 3D CT; (**C**) Post-operative frontal view of 3D CT; (**D**) Pre-operative profile view of 3D CT; (**E**) Pre-operative design of profile view of 3D CT; (**F**) Post-operative profile view of 3D CT.

Eleven patients with 15 ankylosed joints were included in this study (Table 1, Figs 1, 2, 3, 4, 5, 6, 7, 8, 9). There were 3 males and 8 females with a mean age of 45 years (from 27–62 years). The mean duration of ankylosis was 27 years (from 3–50 years). Three cases were caused by infection and eight were caused by trauma. There were 7 unilateral cases and 4 bilateral cases. Eight patients had undergone multiple previous surgeries, including autogenous bone grafts or gap arthroplasties. Among them, 5 patients had 1 previous surgery, 2 patients had 2 previous surgeries, and 1 patient had 3 previous surgeries.

Four patients with bilateral TMJ ankylosis with severe mandibular deficiency had their mandibular rami elongated by the prosthesis. Among them, 2 had simultaneous LeFort I osteotomies (Figs 7, 8, 9.). The mean follow-up period of the 11 patients was 21 months (from 12–31 months). There were no reports of infection, breakage, or loosening of the prostheses. Mouth opening was significantly improved from 5.5 mm preoperatively to 31.6 mm postoperatively (Table 1, p < 0.05). CT scans during at least 6 months follow-up after surgery indicated that there was no bone resorption around the screws and no ectopic bone formation around the artificial condylar heads. In the 4 patients with mandibular ramus elongation, there was no significant change in the SNA angle after operation (p > 0.05, Tables 2, 3) but the SNB angle significantly improved from 61.99° before operation to 67.86° after operation (p < 0.05). Ramus heights were significantly elongated 7.25 mm on the right side and 8.50 mm on the left side after operation (p < 0.05). The mean chin advancement was 10.19 mm.

## Discussion

Temporomandibular joint reconstruction is an effective way to treat TMJ ankylosis without residual condyles. Autogenous bone grafts, alloplasticTMJ prostheses, and distraction osteogenesis are 3 methods that can be employed for TMJ reconstruction. Alloplastic TMJ prosthesis is widely used in Europe and North America for a variety of TMJ maladies. Compared to autogenous bone grafts, it has advantages, such as stability, and does not require a second operative region, which reduces surgical trauma and shortens operative time. Alloplastic TMJ prostheses are divided into 2 types: standard and individualized. In 2013, Leandro reported a 10-year follow-up of 300 patients using Biomet standard prostheses. These significantly improved mouth opening and reduced pain scores, making them a safe and effective method of TMJ reconstruction^14^. In 2015, Wolford reported a 20-year follow-up of 56 patients treated with TMJ Concepts individualized prostheses^15^. These also enhanced mouth opening and mandibular movement, relieved pain, and helped improve patients’ diet. About 85.7% of patients reported improved quality of life. There was no prosthesis abrasion, breakage, or failures that required removal. Although there are reports using TMJ prostheses to treat jaw deformity, most of them are customized device which are much easier than standard ones to implant. For ankylosis patients with severe jaw deformity, simultaneous treatment is sparse because first the ankylosis has to be released for the patients to open mouth widely and then jaw deformity can be corrected in a second operation. In this article, we used CAD/CAM technique to guide ankylosis removal, severe jaw deformity correction and Biomet standard TMJ prostheses implantation simultaneously. Because of the use of digital guides and occlusal splints, there was no need for a second surgery to place the TMJ prostheses. The Biomet fossa prostheses can be stabilized without bone grafts in ankylosis patients with plenty of bony fusion in the glenoid fossa. However, when treating patients with large mandibular angles or mandibular asymmetry, it is very difficult to correctly place the Biomet TMJ prosthesis because of its limited angle design. Extensive bone trimming in the condylar neck area and bone grafting in the mandibular angle area were needed for a stable prosthesis placement without dislocation. Virtual computer surgery illustrated the exact areas requiring bone trimming and grafting and the location of the prosthesis. Digital guides helped transfer this plan in the operation. The results are stable. We had no malocclusion or prostheses dislocation in these patients.

Thus far, there have few studies on the use of alloplastic TMJ prostheses to treat ankylosis. Mercuri^6^ and Wolford^9^ reported using TMJ Concepts (Ventura, CA) individualized prostheses combined with abdominal free fat graft for treatment of TMJ ankylosis. Mouth opening was significantly improved after at least 1 year of follow-up. Compared to other studies without using free grafts around the artificial condyle^7^,^8^, no recurrence of ankylosis occurred. The difference in our study was that we used a free fat graft from the submandibular incision instead of harvesting from the abdomen, which avoided a secondary incision and reduced surgical trauma. Research shows that free fat grafts can prevent blood clot formation and osteogenesis in the osteotomy gap, thus effectively preventing recurrence of ankylosis^6^,^9^. In our study, no malocclusion or recurrence of ankylosis occurred, possibly because of the benefits of digital medicine and free fat grafts.

Compared to autogenous bone grafts, the advantages of alloplastic TMJ prostheses are stability and low ankylosis recurrence rates. Our previous studies on autogenous bone grafts, including rib grafts and coronoid process grafts for treatment of ankylosis, showed that there were no significant differences of mouth opening among rib grafts, coronoid process grafts, and alloplastic TMJ prostheses^16^. However, both rib grafts and coronoid process grafts reported bone resorption and recurrence of TMJ ankylosis^16^. In this study, we used alloplastic TMJ prostheses combined with free fat grafts, and no bone resorption around fixation screws or ectopic bone formation around artificial condyles were reported after a mean follow-up of 21 months. Zhang’s research revealed that glenoid fossa injury plays an important role in the development of TMJ ankylosis^17^. We also found ectopic bone formation from both the fossa and the stump of the ramus in the recurrent patients treated by autogenous bone grafts^16^. This may be due to the fact that exposure of the glenoid fossa and stump of the ramus after osteotomy stimulates rapid bone formation. The reasons for the low recurrence of ankylosis after alloplastic TMJ prostheses may be as follows: first, the fossa prosthesis covers the exposure of the bone and prevents downward bone formation by acting as a barrier; second, the stability of the osteotomy gap is maintained by the mandibular prosthesis; and third, the interposition of the free fat graft around the artificial condyle prevents ectopic bone formation.

Another advantage of alloplastic TMJ prostheses is that they can bear more stress than autogenous bone grafts without resorption, especially in elongating the mandibular ramus to simultaneously correct mandibular deficiency. It therefore is an effective method for treating TMJ ankylosis combined with mandibular deformity in adults. In this study, we used alloplastic TMJ prostheses to elongate the mandibular ramus in 4 patients; among them, 2 were combined with LeFort I osteotomy. The results showed their SNB, ramus height, and chin advancement were significantly improved after operation. Although TMJ prostheses can provide stable results, when combining with orthognathic surgery, preoperative design and postoperative orthodontic treatment are highly demanding especially for recurrent TMJ ankylosis. The scarring from previous surgery may inhibit advancement of the mandible. Both an ideal and a palliative plan should be considered before operation. Discussion with orthodontists before operation should be performed to ensure the best jaw position and to facilitate postoperative orthodontic treatment. For patients with gross mandibular advancement, Biomet standard TMJ prostheses may present a shortage of shapes and lengths. Individualized TMJ prostheses may fit those patients better. The surgical sequence is also different from the routine orthognathic surgery. Because of mouth opening limitations, TMJ ankylosis should be relieved first, allowing placement of an occlusal splint to position the mandible prior to insertion of the TMJ prosthesis. The Le Fort I osteotomy should be performed last. The operation must strictly obey aseptic principles to avoid infection from the mouth. Two sets of instruments should be used separately for extra-oral and intra-oral operations. Surgical gowns and gloves should be changed, with disinfection and redraping, when moving the surgical field from the mouth to the TMJ area. Antibiotics should be used for1 week after surgery to prevent infection.

In conclusion, the use of standard alloplastic TMJ prosthesis is a reliable method to treat TMJ ankylosis, especially in recurrent cases. By CAD/CAM technique, this procedure can correct jaw deformity simultaneously with stable results.

## Patients and Methods

### Patient selection

This was a retrospective study on the use of Biomet prostheses for TMJ reconstruction in patients with TMJ ankylosis. It was approved by the Independent Ethics Committee of the 9^th^ People’s Hospital. We confirm that all methods were performed in accordance with the relevant guidelines and regulations. The authors had access to information that could identify individual participants during or after data collection. Patients with TMJ ankylosis treated from January, 2013 to December, 2015 in the Department of Oral Surgery, Shanghai Ninth People’s Hospital were reviewed. Informed consent was obtained from all patients. The inclusion criteria were: 1.① Age ≥ 18 years old; 2. No residual condyle present on the medial side of the bony fusion; 3. TMJ reconstructed using Biomet standard prostheses; 4. Patients had CT data before operation, 1 week after operation, and at least 6 months after operation. The exclusion criteria were: 1. Residual condyle present on the medial side of the bony fusion; 2 TMJ reconstruction without Biomet standard prostheses; 3. Incomplete clinical and CT data.

## Method

Mouth opening, occlusion, and facial appearance (deviation, retrusion) of all patients were recorded preoperatively. Maxillofacial CT scans (slice thickness, 1 mm; reconstruction thickness, 0.625 mm; GE, America) were taken before operation, within 1 week after operation, and at least 6 months after surgery for all patients. Their data were imported into Proplan CMF 1.4 Software (Materialize, Leuven, Belgium) as Digital Imaging and Communications in Medicine (DICOM) and used to perform coronal and three-dimensional (3D) reconstruction of jaw bones. The depths and ranges of TMJ bony fusions were measured in the coronal and 3D reconstructions. After virtual bony fusion removal, TMJ prostheses were virtually placed for best position. Osteotomy and prosthesis location guides were designed and fabricated by 3D printing^10^. For patients with severe mandibular deficiency, after defining the facial midline and Frankfort plane, SNA (sella, naison, and supspinale angle), SNB (sella, naison, and supramentale angle), the heights of the ramus (distance between the gonion and zygomatic root point), and chin retrusion (distance between the gnathion and perpendicular line through N to the FH plane) were measured (Fig. 1). TMJ prostheses were used to elongate the mandibular ramus when necessary. LeFort I osteotomy was combined simultaneously according to the severity of jaw deformity. Digital occlusal splints were used to assist the prostheses placement^13^.

Modified preauricular and submandibular incisions were used to expose the bony fusion and ramus. The osteotomy guide was used to assist bony fusion removal (Fig. 2). The ipsilateral coronoid process was also removed. Then, the extraoral incision was isolated from the oral cavity. Intraoral disinfection was performed. The contralateral coronoid process was removed intraorally according to the degree of mouth opening to ensure it was ≥40 mm. Intermaxillary fixation was performed using an interocclusal splint, if necessary (Fig. 3). Subsequently, the oral cavity was sealed, and the facial area was disinfected and again redraped. Surgical gowns and gloves were changed. Glenoid fossa and mandibular ramus prostheses were positioned and stabilized with the help of surgical guides. At least 4 screws were inserted for both the fossa and ramus components. The artificial condylar head was checked to ensure that it was located in the posterosuperior position of the fossa component. The osteotomy gap was filled with free fat taken from the submandibular incision. Two drains were placed inside the surgical incision after copious chloramphenicol flushing. For patients with severe jaw deformities, a LeFort I osteotomy was then performed.

Antibiotics were used intraoperatively and postoperatively for 1 week. CT scans were taken to check bony fusion removal and TMJ prosthesis positioning by coronal and 3D reconstruction within 1 week after surgery. No intermaxillary fixation was needed after operation. With at least 6 months of follow-ups, mouth opening and facial contour of the patients were recorded and compared with those before the operation. CT scans at follow-ups were taken to check prosthesis stability, whether there was a breakage in prosthesis or bone resorption around screws, and ectopic bone formation.

For patients with mandibular deficiency, SNA, SNB, ramus height, and chin advancement were measured and compared before operation and during follow-ups.

### Statistical analysis

Data were analyzed using the Statistical Package for Social Sciences software package, version 17.0 (SPSS, Chicago, IL). Mouth opening, SNA, SNB, ramus height, and chin advancement before and after operation were compared using the paired t-test of 1-way analysis of variance. An α level of ≤0.05 was considered significant.

## Additional Information

**How to cite this article:** Hu, Y.H. *et al*. Simultaneous treatment of temporomandibular joint ankylosis with severe mandibular deficiency by standard TMJ prosthesis. *Sci. Rep.* **7**, 45271; doi: 10.1038/srep45271 (2017).

**Publisher's note:** Springer Nature remains neutral with regard to jurisdictional claims in published maps and institutional affiliations.

## Figures and Tables

**Table 1:** Information of the TMJ ankylosis patients treated by Biomet TJR.

No.	Gender	Age (year)	Duration (year)	Cause	Follow-up (month)	Sides	MIO (mm)	
							Pre	Post
1	Female	57	50	Trau	31	Uni	10	33
2	Female	62	50	Infe	30	Uni	0	35
3	Female	35	3	Trau	20	Uni	3	35
4	Female	32	18	Trau	28	Bi	18	27
5	Male	39	25	Trau	24	Bi	3	35
6	Male	27	5	Infe	26	Uni	0	35
7	Female	39	30	Trau	23	Uni	8	28
8	Male	42	34	Trau	14	Bi	15	25
9	Female	51	35	Infe	16	Uni	1	35
10	Female	62	8	Trau	12	Uni	0	35
11	Female	52	40	Trau	8	Bi	3	25
Mean		45.3	27.09		21.09		5.55*	31.64*

Abbreviations: MIO, maximal incisor opening; Trau, trauma; Infe, infection; Uni, unilateral; Bi, bilateral *p = 0.000

**Table 2:** Jaw changes of 4 patients with bilateral mandibular ramus elongation.

No.	SNA(°)		SNB(°)		Ramus height (mm)				Distance of chin retrognathia (mm)		
	Pre	Post	Pre	Post	Right		Left		Pre	Post	advancement
					Pre	Post	Pre	Post			
1	78.82	74.01	62.46	70.89	54.50	64.25	54.66	64.31	38.29	19.68	18.61
2	75.63	79.13	58.07	64.4	64.45	67.82	63.92	68.73	48.9	41.7	7.20
3	77.28	77.33	66.17	71.82	67.43	71.63	63.91	69.16	34.39	26.92	7.47
4	80.73	79.19	61.23	64.31	47.6	59.29	46.33	60.63	39.42	31.94	7.48
Mean	78.12	77.42	61.99	67.86	58.50	65.75	57.21	65.71	40.25	30.06	10.19

**Table 3:** P value of the jaw changes.

	SNA(°)	SNB(°)	Ramus height (mm)		Chin advancement (mm)
			Left	Right	
Changes	0.7	5.87	7.25	8.50	10.19
P value	0.356	0.006	0.005	0.004	0.005
